# A method of blasted rock image segmentation based on improved watershed algorithm

**DOI:** 10.1038/s41598-022-11351-0

**Published:** 2022-05-03

**Authors:** Qinpeng Guo, Yuchen Wang, Shijiao Yang, Zhibin Xiang

**Affiliations:** 1 School of Resources Environment and Safety Engineering, University of South China, Hengyang, 421000 China; 2 China Nonferrous Metal Changsha Survey and Design Institute Co., LTD., Changsha, 410000 China

**Keywords:** Civil engineering, Petrology

## Abstract

It is of great theoretical significance and practical value to establish a fast and accurate detection method for particle size of rock fragmentation. This study introduces the Phansalkar binarization method, proposes the watershed seed point marking method based on the solidity of rock block contour, and forms an adaptive watershed segmentation algorithm for blasted rock piles images based on rock block shape, which is to better solve the problem of incorrect segmentation caused by adhesion, stacking and blurred edges in blasted rock images. The algorithm first obtains the binary image after image pre-processing and performs distance transformation; then by selecting the appropriate gray threshold, the adherent part of the distance transformation image, i.e., the adherent rock blocks in the blasted rock image, is segmented and the seed points are marked based on the solidity of the contour calculated by contour detection; finally, the watershed algorithm is used to segment. The area cumulative distribution curve of the segmentation result is highly consistent with the manual segmentation, and the segmentation accuracy was above 95.65% for both limestone and granite for rock blocks with area over 100 cm^2^, indicating that the algorithm can accurately perform seed point marking and watershed segmentation for blasted rock image, and effectively reduce the possibility of incorrect segmentation. The method provides a new idea for particle segmentation in other fields, which has good application and promotion value.

## Introduction

Blasting is widely used in mining and civil engineering due to its economy and efficiency^1–5^. As an important technical indicator of blasting effectiveness, blasted block size distribution directly affects the cost and efficiency of subsequent shoveling, crushing and grinding processes, and also provides a necessary basis for blasting parameter optimization^6–10^. Therefore, it is of theoretical significance and practical value to establish a fast and accurate detection method for particle size of rock fragmentation to guide blasting construction and improve blasting efficiency. Blasted rock piles are characterized by large scale, serious adhesion and irregularly shaped rock clumps, large differences in particle size, and small differences in grayness, which make it difficult to accurately measure the particle size of blasted rocks^11,12^. The existing measurement methods can be summarized into two categories: three-dimensional (3D) point cloud data segmentation measurement and two-dimensional (2D) image segmentation measurement. The 3D point cloud data is mainly obtained using 3D laser scans or a large number of high-resolution digital photos taken by Unmanned Aerial Vehicle (UAV)^13,14^. Han and Song^15^ used stereophotogrammetry for 3D modeling of blasted rock piles to obtain surface block dimensions and corrected for errors in rock fast due to stacking by indoor tests, and validated the applicability of the method in small-scale tests in the field. Although the creation of point cloud data using stereophotogrammetry is more effective and cheaper, the large number of images received takes a lot of time to process and convert into point cloud data^16^. 3D laser scanners are widely used in mine surveying due to its directly, quickly and captures 3D geometry in detail^17–21^. Engin et al.^22^ used a 3D laser scanner to obtain a 3D view of a rock piles of about 13 cm and used morphological methods to determine the position of the rock block and to correct the surface of the rock block, and finally used nonlinear order statistical filtering and histogram analysis to determine the blasted block size distribution of the rock piles, and by comparing the results of image analysis, the results obtained using this method were proved to be sufficiently reliable and accurate. Wang et al.^23^ used 3D laser scanning technology to obtain blasted rock piles point cloud data, and used the Voxel Cloud Connectivity Segmentation algorithm improved by discrete features to solve the influence of point clouds on the surface of small particles of ore on block recognition, and used the Locally Convex Connected Patches algorithm improved by plane fitting to solve the problem of over-segmentation of large rock blocks, and verified the generality and accuracy of the method by comparing the number of rock blocks. The methods for rock segmentation measurement of 3D point cloud data are mainly clustering analysis or converting point cloud data into similar 2D images and then using 2D image segmentation methods^21,23,24^. Compared with the 2D image rock segmentation measurement method, its main advantage is the high accuracy of the obtained point cloud data, but due to the expensive 3D laser scanner and the need for professional software for pre-processing, it cannot be widely used.

The 2D image segmentation measurement method has been used for nearly 30 years, which is currently the most used method for blasting fragmentation size measurement. Researchers studying and applying this method have also developed various commercial software, such as Wipfrag and Gold Size developed in Canada, Split and Cias developed in the United States, Ipacs and Kth developed in Sweden, Fragscan developed in France, Tucips developed in Germany, Power Sieve developed in Australia, and India developed Fragalyst 3.0, of which Wipfrag and Split are the most commonly used commercial software^25–30^. According to different principles, image segmentation methods are divided into four main categories: threshold segmentation, region segmentation, edge segmentation, and artificial neural network segmentation methods^31–37^. Among them, the most applied methods in rock segmentation are region and artificial neural network segmentation methods. Li et al.^38^ used the GAN-Unet model to segment images on the ore delivery belt, and the results showed that the method can reduce the problems of unclosed edges, over-segmentation, and under-segmentation, and improve the graph segmentation accuracy. Liu et al.^39^ first extracted the contour of the conveyor belt ore image using the U-Net model and binarized the image, and then used the Res_UNet model for contour optimization. The results of segmentation show that the model based on U-Net and Res_UNet is more suitable for conveyor belt ore image segmentation compared with the watershed algorithm and the U-Net model without contour optimization. However, the training time of the Neural network for image segmentation method is extremely long, and the above study is aimed at the conveyor belt ore with small particle size differences, and whether it applies to the segmentation of large-scale blasted rock blocks with large particle size differences needs to be studied. Yang et al.^40^ adopt a new affinity image construction model to improve the superpixel image segmentation technique for rock block segmentation. DexiNed edge detection network was introduced by Li et al.^41^, and implemented rock block segmentation using morphological transformation and watershed algorithm to avoid the influence of noise in binary images on rock block segmentation. Li et al.^42^ adopt binarization of rock block images using the U-Net model and segmentation of cohesive rock blocks using the watershed algorithm to solve the mis-segmentation of rock blocks caused by small differences in rock block grayness, and the results showed that the method is high robustness and high accuracy.

The watershed algorithm is widely used for rock particle image segmentation because of its good response to weak edges^43,44^. Ma et al.^45^ introduced an improved algorithm based on distance transform and morphological gradient. The result proved that the method is effective, accurate and rapid, which basically meets the requirements of unsupervised automatic acquisition of ore granularity parameters. Yao et al.^46^ improved the watershed algorithm using local minimum with threshold, effectively segmenting adhesive grains while avoiding over-segmentation.

To realize the accurate and rapid segmentation of blasted rock block image, the image pre-processing process and the impact on the segmentation results are studied first in this study, and then the causes of redundant seed points generated by the watershed algorithm in the process of blasted rock block image segmentation are analyzed. To explore the new automatic marking method of seed points, the shape characteristics of blasted rock blocks are analyzed and introduced into the watershed algorithm, a seed point marking method based on the solidity of rock block contours is proposed, and finally, an adaptive segmentation algorithm for blasted rock blocks based on block shapes is proposed based on the distance-based optimized watershed algorithm. The important contributions of this study are as follows.

1. Blasted rock block contours have a high solidity.
2. An automatic seed point marking method based on the solidity of rock block contour is proposed.
3. An adaptive segmentation algorithm for blasted rock blocks based on block shapes is proposed.

## Image acquisition

**Figure 1:**
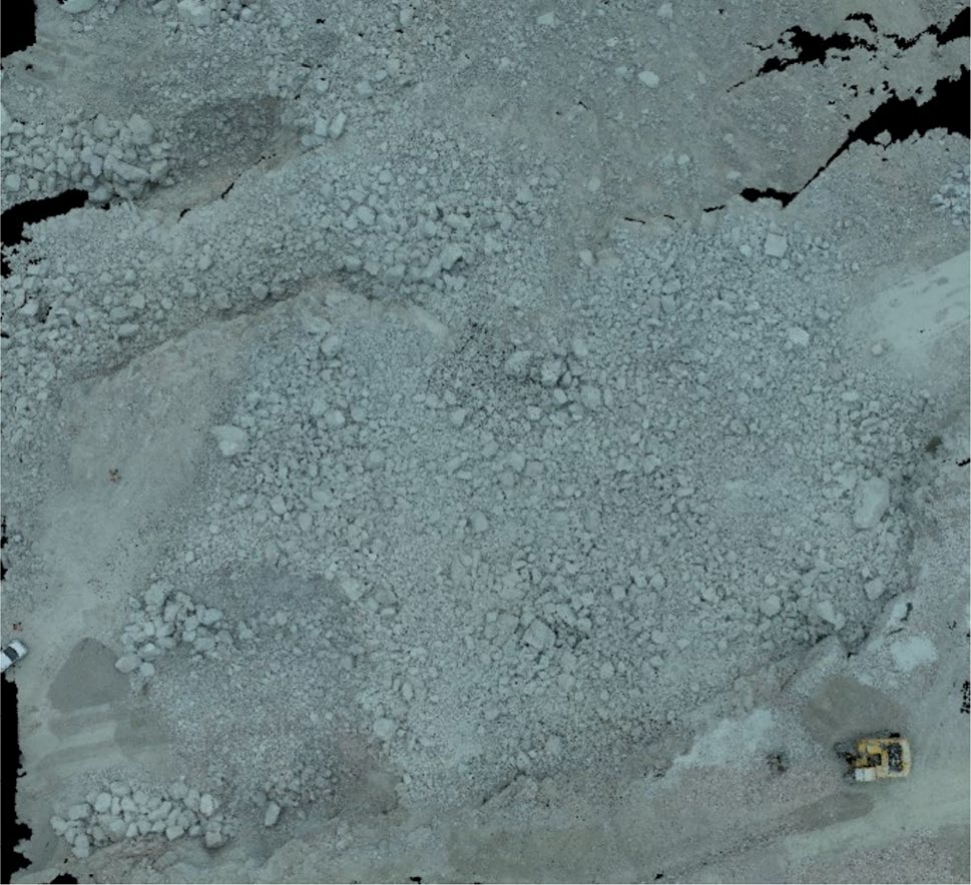
3D view of limestone quarry after blasting.

**Figure 2:**
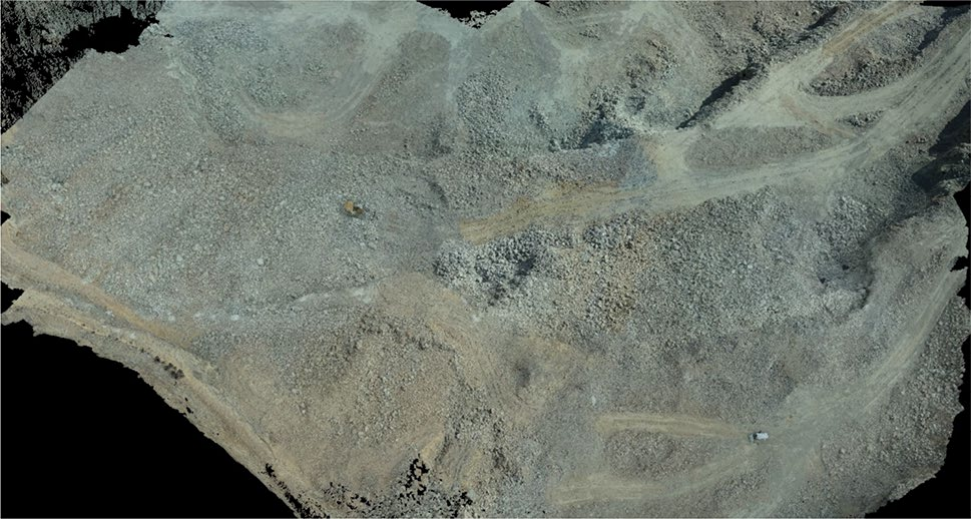
3D view of granite quarry after blasting.

Limestone quarries and granite quarries located in Huizhou, China were selected for the study, are both operated by Guangdong Xiyuan Blasting Technology Co., Ltd. Therefore, the blasting design parameters are the same, except for the charge quantity. The step height of the quarry is about 12, the diameter of the gun hole is 140 mm, using a digital electronic detonator to hole-by-hole detonation. Figures 1 and 2 show the 3D images established by aerial photography of a limestone quarry and a granite quarry after a certain blast using a UAV, respectively.

**Figure 3:**
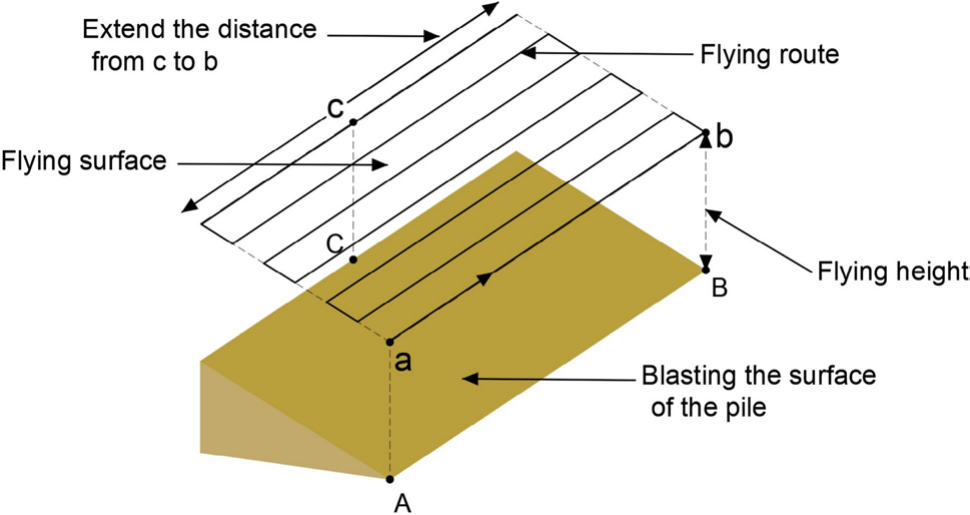
UAV flight image.

**Figure 4:**
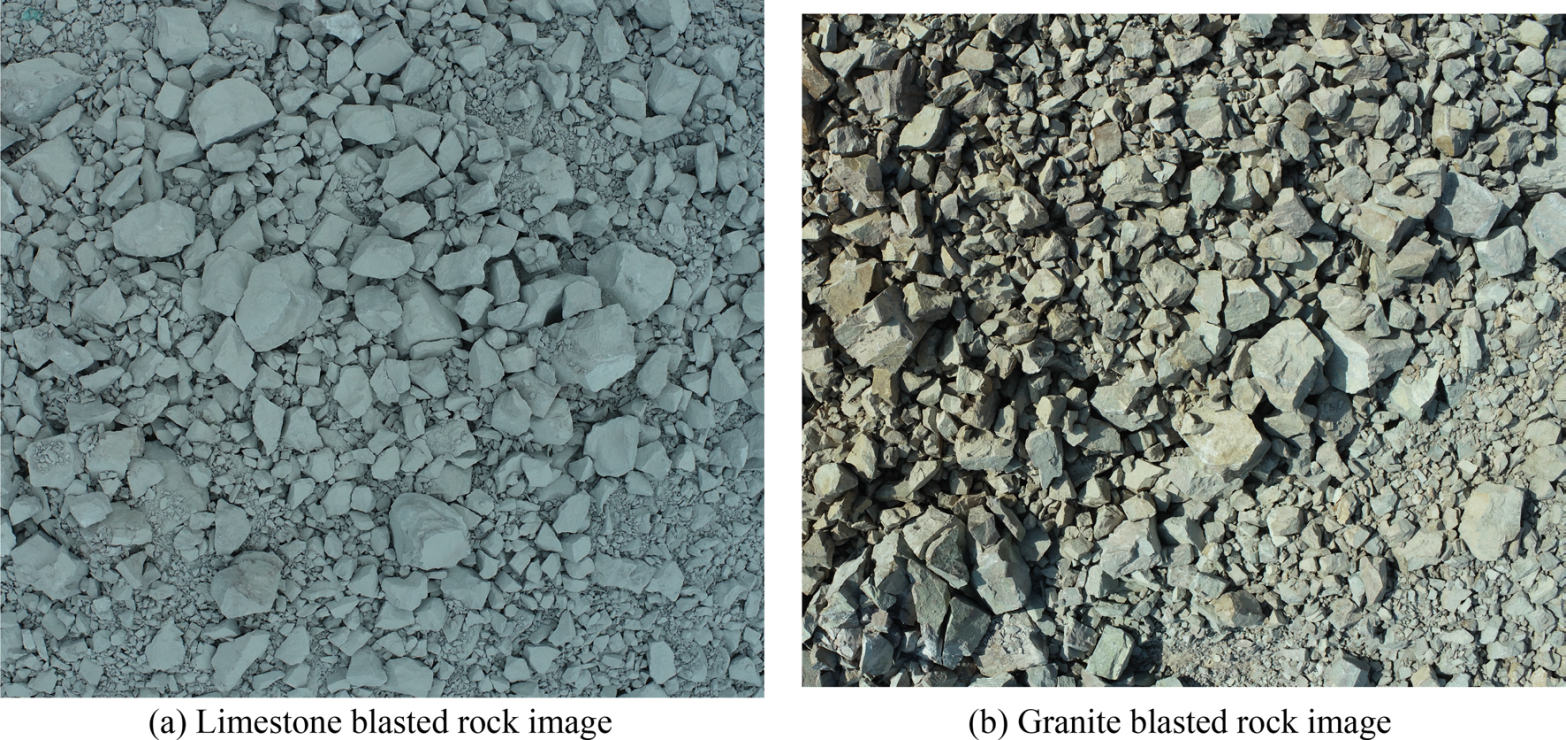
Blasted rock images acquired by UAV.

The UAV used the DJI Phantom 4 RTK, which is equipped with a multi-frequency and multi-system RTK module. The camera is synchronized with the RTK module $\mu s$-level time to provide real-time $\mathrm{cm}$-level positioning data without the need to deploy image control points. The UAV is equipped with a high-precision anti-shake tripod head camera that supports up to 20 megapixels of still photo shooting. To avoid errors caused by rock block overlap, image acquisition of blasted rock piles is performed using tilt photogrammetry, where the camera orientation is perpendicular to the blasted rock piles surface. Figure 3 shows the flight schematic of the UAV. Firstly, determined point A, B and C. Then, the outward expansion was carried out from point C to A and B respectively until the flight range completely covered the rock piles, and the UAV automatically planned the flight route according to the overlap rate. To ensure clarity in images, the flight altitude was set to 25, which is the minimum flight altitude of the UAV. The collected images of limestone and granite blasting blocks are shown in Fig. 4.

## Proposed methods

### Image pre-processing

**Figure 5:**
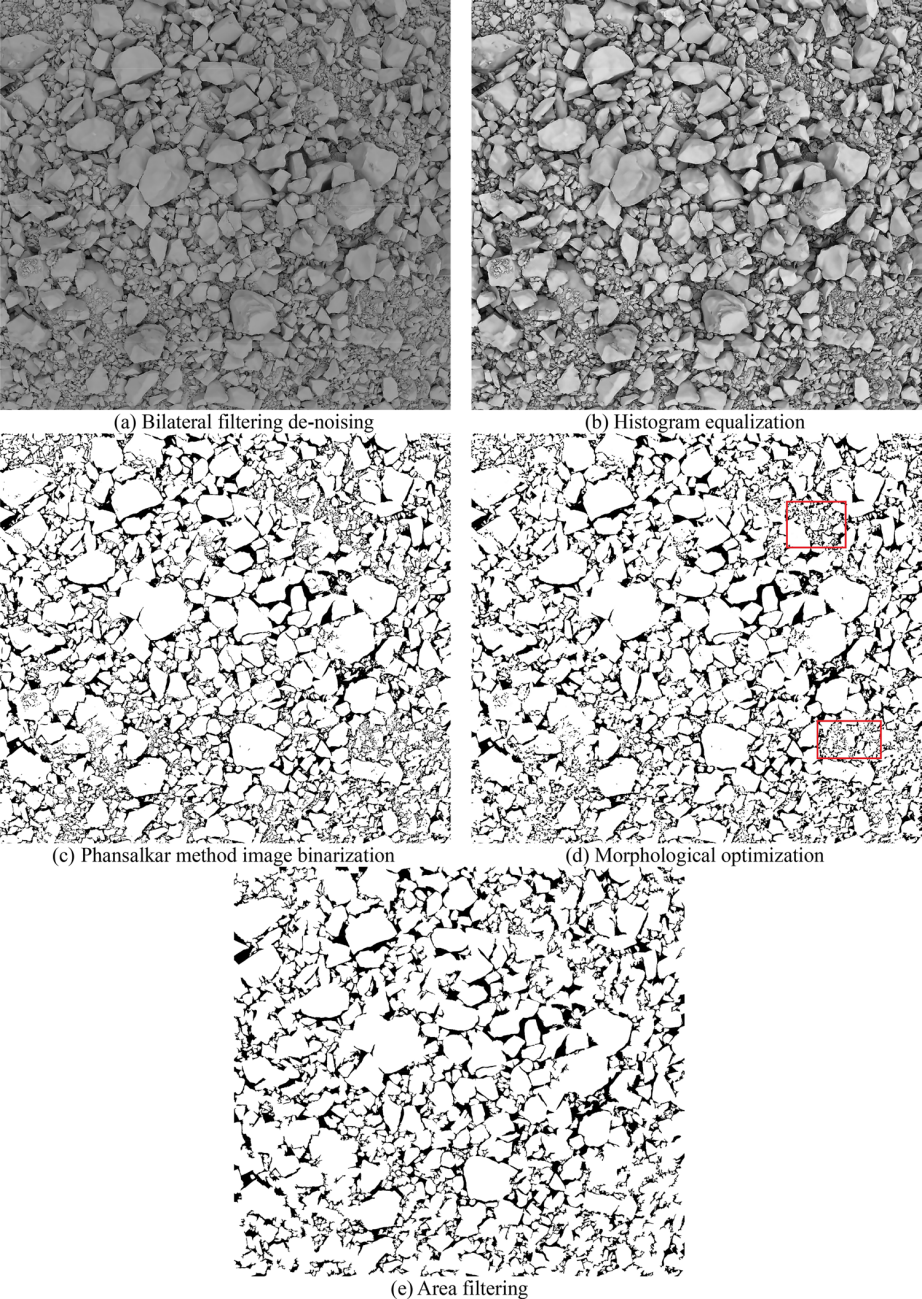
Image pre-processing effect of limestone blasting block.

Due to the more complex and dustier environment of the quarry, there is more serious noise in the images of blasted rock piles collected through the camera, and the rock piles are heavily stacked and adhered to each other, with small background difference degree and inconspicuous color information. To effectively segment the rock blocks, pre-processing is required for the blast rock piles images. There are many methods of image pre-processing^47,48^, and this study adopts the more commonly used methods in the field of rock segmentation. Firstly, the grayscale image is de-noising by bilateral filtering. Secondly, the rock block edges are made more visible by limiting the contrast adaptive histogram equalization, then the image is binarized using the Phansalkar method^49^ based on local image properties. Finally, the binary image is morphologically optimized and area filtered. Figure 5 shows the effect of limestone blasting rock block image after pre-processing.

The Phansalkar method^49^ is an adaptive local thresholding method based on local image properties, which processes each pixel $(x,y)$ in an image by considering a $w\times w$ window with the pixel as the center on that window with a grayscale mean is $m(x,y)$ and standard deviation is $s(x,y)$, then the local threshold $T(x,y)$ for the pixel is:

1 $$T\left(x, y\right)=m\left(x, y\right)\left[1+p{e}^{-qm\left(x,y\right)}+k\left(\frac{s\left(x, y\right)}{R}-1\right)\right],$$

where $p$ and $q$ are constants. The Phansalkar method is flexible in that it determines the selection of the threshold value based on the magnitude of the local mean and the standard deviation, and by adjusting the values of the parameters $p$, $q$ and $k$, different processing results are obtained. the processing effects are shown in Fig. 5c.

**Figure 6:**
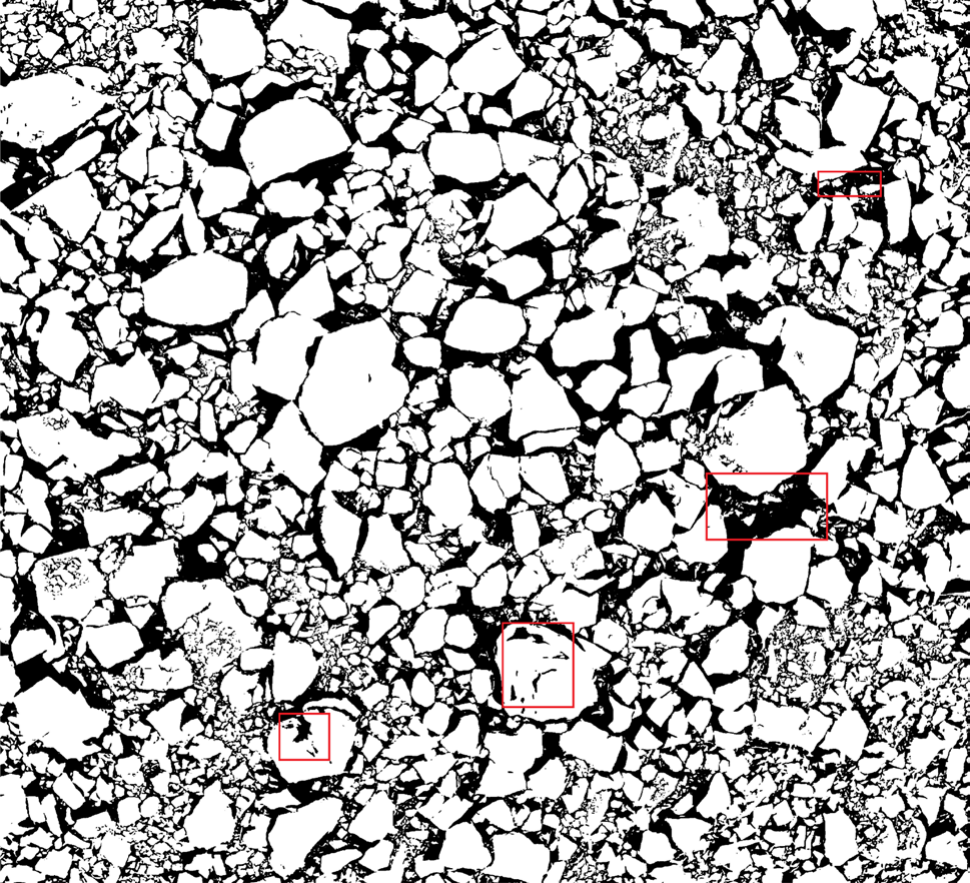
Otsu method image binarization.

To show indicate the effectiveness, the method is compared with the Otsu method^50^, and the results of the processing of Fig. 5b using the Otsu method are given in Fig. 6, from which it can be seen that the Otsu method produces three kinds of errors: (1) incorrectly dividing regions into backgrounds, such as darker regions and zones obscured by shadows; (2) incorrectly dividing small rocks into backgrounds; (3) dividing part of the inner regions of large rocks into backgrounds. Some of the error areas are shown in the red box part of the image, which can seriously affect the subsequent rock identification. The Phansalkar method can accurately distinguish the background from the rock particles with satisfactory results.

Figure 5d shows the binary image of the blasted rock piles after morphological optimization. Comparing this image with Fig. 5c, we can see that morphological optimization can eliminate the small “holes” and noise points in the binary image and smooth the target edges, but some of them still cannot be completely removed. Figure 5e uses the area filtering elimination method to achieve more satisfactory results, but there are still some noise points, which affect the rock segmentation. When the area threshold increases, it causes some edges to be removed. As in the red box in Fig. 5d, small rock block contour are eliminated, which has an impact on the segmentation results.

### Principle of watershed segmentation algorithm based on distance transformation

**Figure 7:**
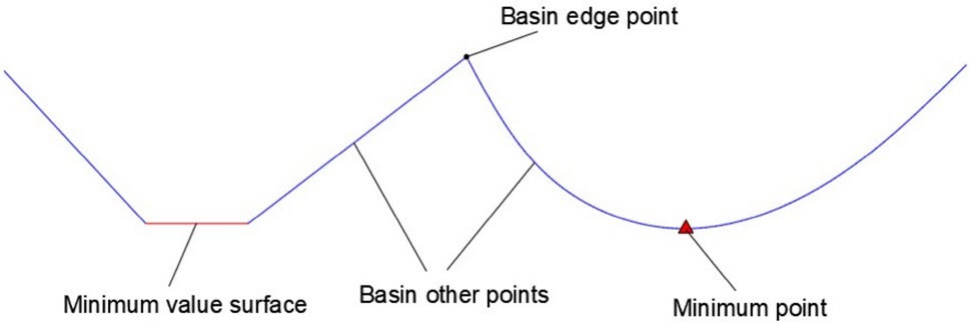
2D topographic image of watershed.

The principle of the watershed algorithm is to visualize an image as a 3D topographic image^34^. In the terminology of “topography”, three types of points are considered. In Fig. 7: ① a local min point (min value surface), which corresponds to the lowest point of a basin; ② points at other locations in the basin; ③ points at the edge of the basin, where the basin meets other basins. For a specific regional minimum, the set of points meeting condition ② is called the catchment basin or watershed for that minimum, and the points meeting condition ③ form the front line of the ground called the division line or watershed line. The main goal of the algorithm is to find the watershed line, which is the contour of the rock block image. Assuming that a hole is punched at the minimum of each area, we let the water pass through the hole to flood the entire terrain at a uniform rate. As the water rising in the different catchment basins aggregates, a dam is built to stop this aggregation until the water floods the highest point of the topographic image, and the boundaries of these dams are the dividing lines of the watershed.

The traditional watershed algorithm is usually marker less segmentation, and the input object is a gradient image, which is based on the luminance change, and it only reflects the edge information of the image, which will result in unreasonable segmentation because of its noise-sensitive feature, leading to the following disadvantages of the watershed algorithm: ① the noise in the original image causes the segmentation contour shift; ② the image with low contrast, the contour of the target region is easily lost when segmenting; ③ there are many meaningless local minima in the image. Therefore, the most common practice is to perform a distance transformation on the binary image.

The distance from each pixel in a binary image to its nearest zero-valued pixel is called the distance transform^51^. Suppose a binary image I with a target set O and a background set B and a distance image D. The distance transformation is defined as in Eq. (2).

2 $$D\left(p\right)=\mathrm{min}\left(disf\left(p,q\right)\right) p\in O,q\in B.$$

The Euclidean distance is generally chosen as the $disf()$. The calculation method is as follows:

3 $$disf\left(p\left({x}_{1},{y}_{1}\right), q\left({x}_{2},{y}_{2}\right)\right)=\sqrt{{({x}_{1}-{x}_{2})}^{2}+{({y}_{1}-{y}_{2})}^{2}.}$$

### Defects of watershed segmentation algorithm based on distance transformation

**Figure 8:**
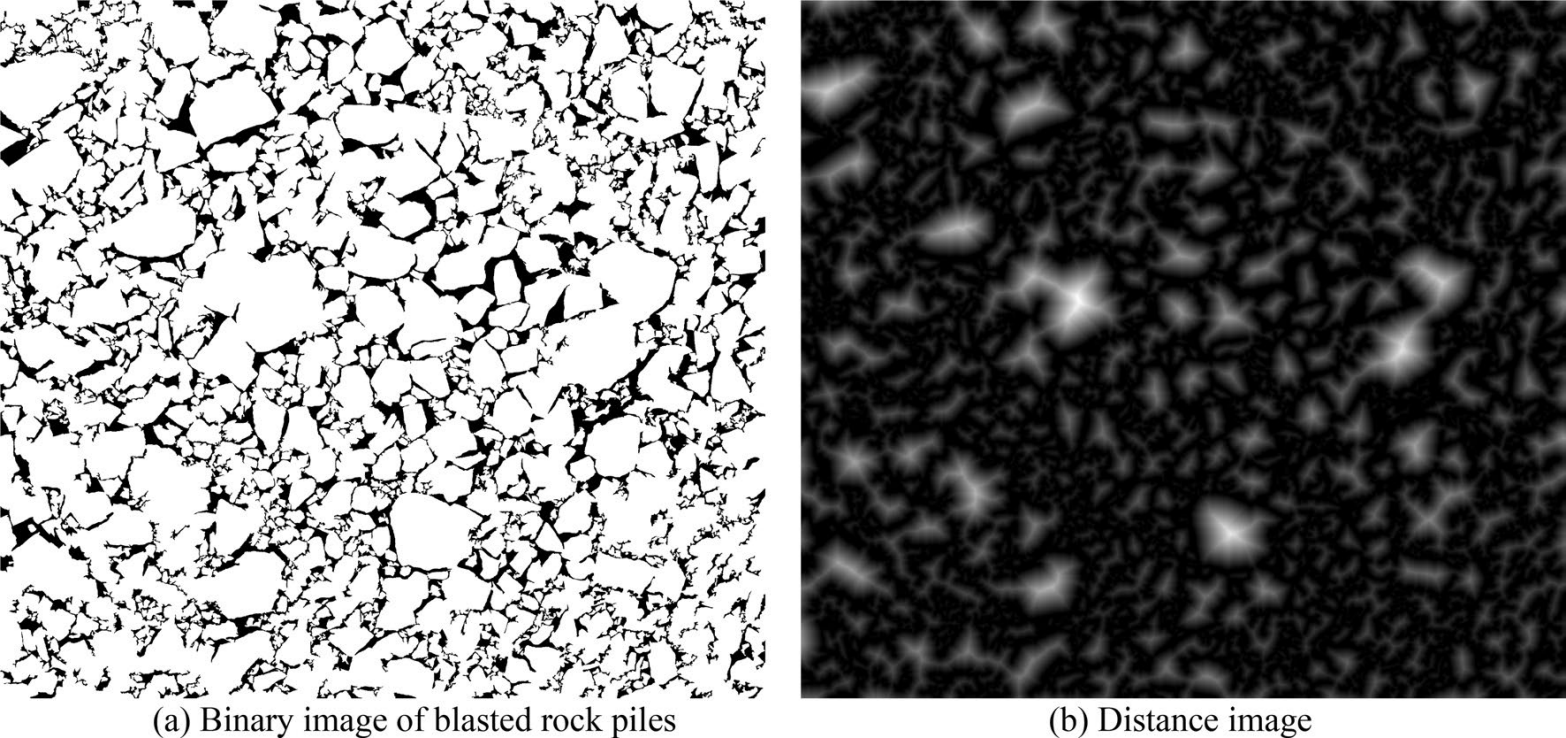
Distance transformation.

Figure 8 gives the distance transformed image of the binary image of the blasted rock piles. From Fig. 8b, the distance image of the binary image is similar to image skeletonization and still retains the general shape.

**Figure 9:**
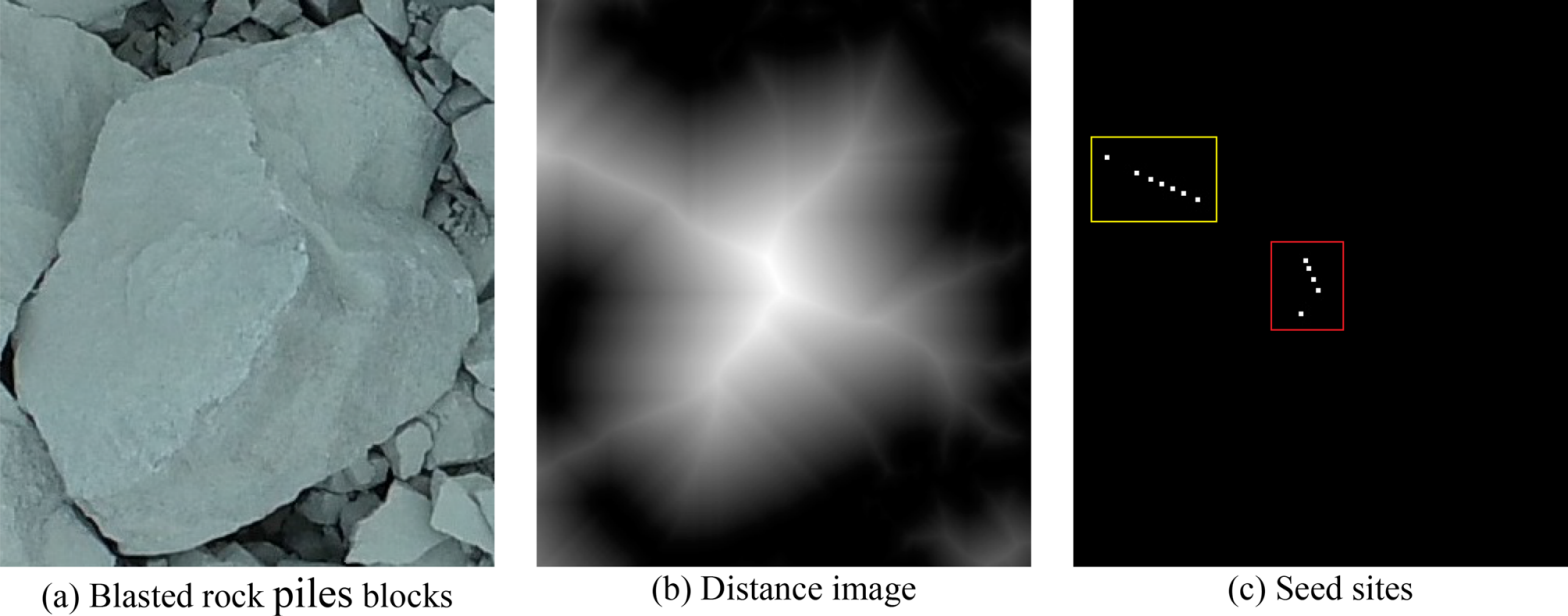
Detailed image of distance transformation and seed point image of rock block.

Figure 9 shows the distance transformation detail image and seed point image of a rock block, where (a) is a rock block in the image; (b) is the distance transformation image; (c) is the local extreme value point (seed point). From Fig. 9c, it can be seen that there are multiple extreme value points, i.e., redundant seed points, in the center of the block (in the red box), which will cause more serious over-segmentation, and there are also more extreme value points in the sticky part of the two blocks (in the yellow box), which seriously affects the segmentation effect of the adhesion block.

For this problem, the commonly used methods are mainly to merge adjacent maximal points and merge expanded maximal points. But as shown in Fig. 9c, the distance between the maximal points inside the rock block and the maximal points in the adhering part of the rock block is large. The above method is difficult to achieve accurate merging. Moreover, for the complex image, the distribution of the maximal points is irregular, and the above maximum point elimination method is less effective.

### Solidity of rock block contour

**Figure 10:**
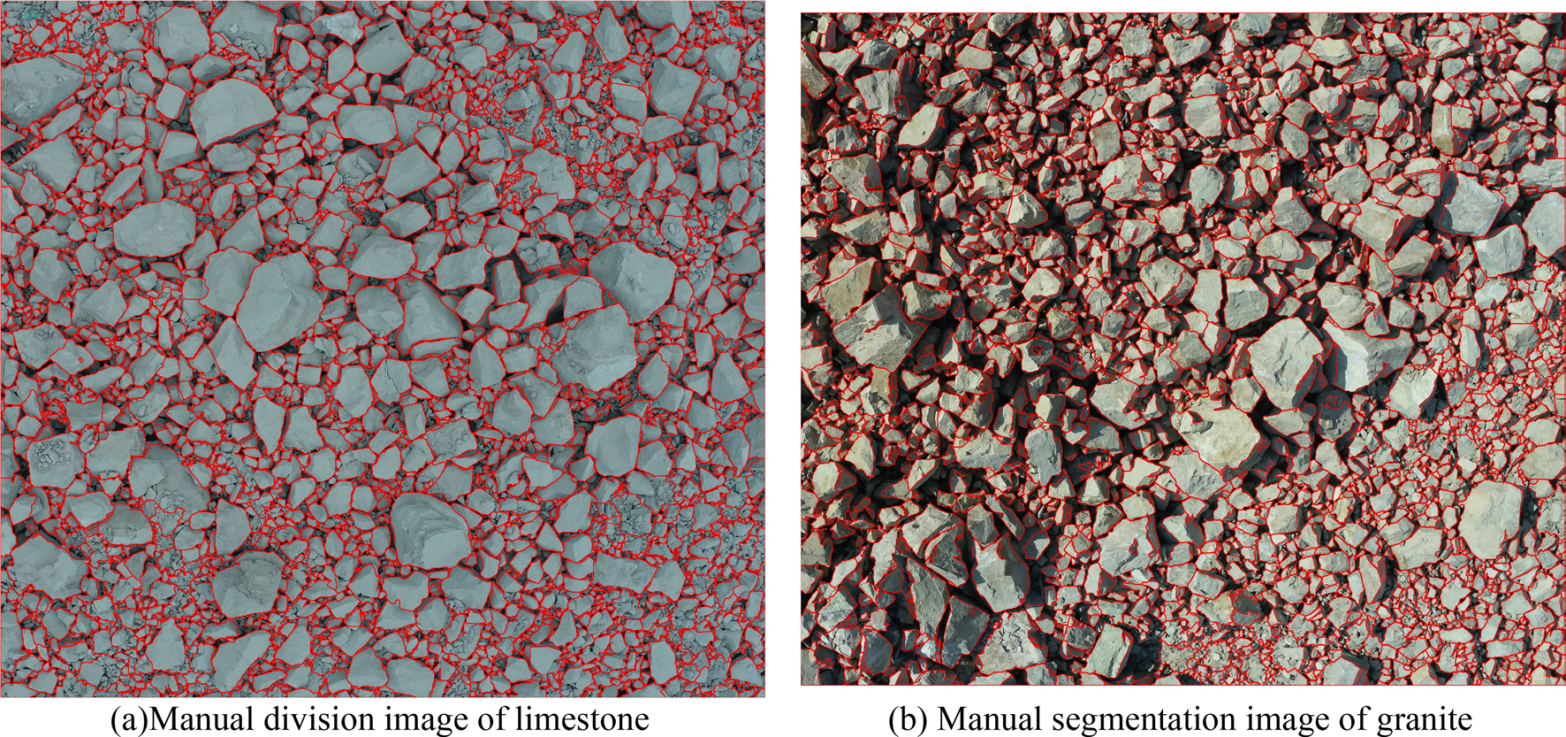
Manual segmentation image.

In the segmentation process of some special adherent objects, such as adherent cells, spherical particles, etc., some prior knowledge, such as the color of cell nuclei, shape of spherical particles and others, can be used in turn to correct the seed points. However, for the segmentation of blasted rock block, there is no a priori knowledge available other than the shape, and the shape of blasted rock block is irregular polygons. To investigate the rule of blasted rock block shape, this study performs manual segmentation of blasted rock images, as shown in Fig. 10.

**Figure 11:**
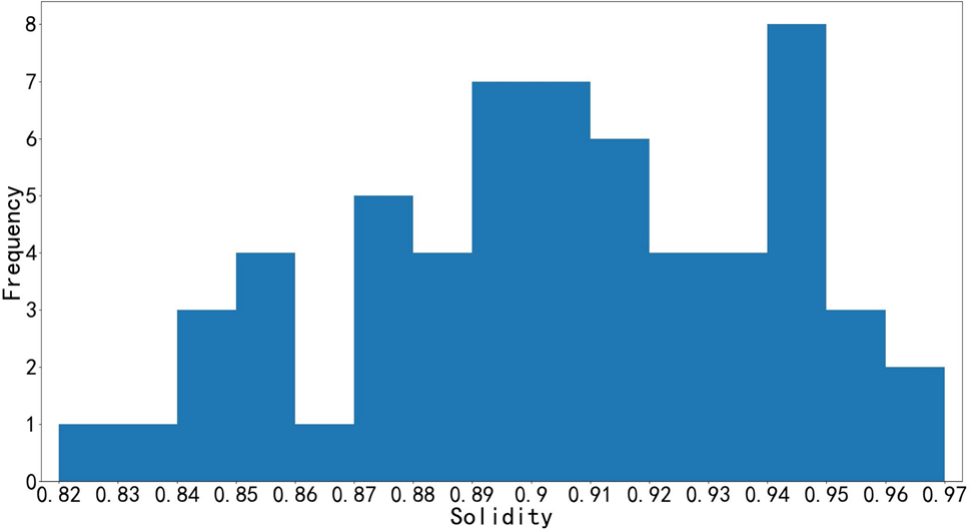
Histogram of limestone solidity distribution.

**Figure 12:**
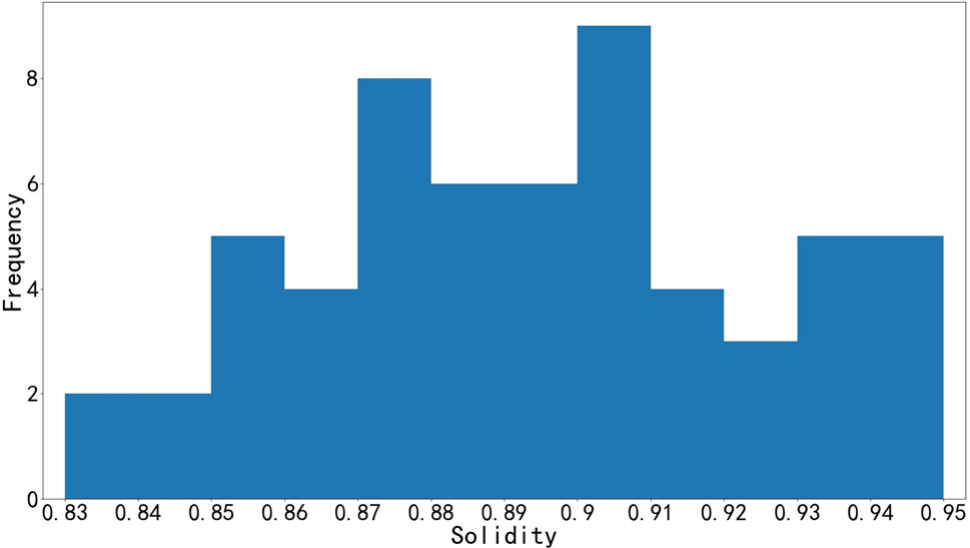
Histogram of granite solidity distribution.

Statistical information of rock blocks, including area, contour convex hull, solidity of rock block, through image contour detection technology, and it should be noted that the solidity is the ratio of area to the contour convex hull. Due to the limitation of space and considering the large segmentation error of small rocks, only part of the information of larger blocks is shown in Tables 1 and 2. The histograms of solidity distribution of limestone and granite blocks are shown in Figs. 11 and 12, respectively.

**Table 1:** Limestone rock block information.

Area cm^2^	Convex hull cm^2^	Solidity	Area cm^2^	Convex hull cm^2^	Solidity	Area cm^2^	Convex hull cm^2^	Solidity
8910	9475	0.94	3039	3487	0.87	2157	2355	0.92
7463	8076	0.92	2949	3148	0.94	2138	2526	0.85
6989	7247	0.96	2879	3007	0.96	2119	2355	0.90
6582	7249	0.91	2849	3237	0.88	1977	2277	0.87
6072	6360	0.95	2824	2986	0.95	1975	2133	0.93
5588	5906	0.95	2733	2868	0.95	1969	2201	0.89
5506	5801	0.95	2624	2884	0.91	1938	2145	0.90
5362	6260	0.86	2585	2860	0.90	1920	2262	0.85
5325	5964	0.89	2575	2947	0.87	1914	2026	0.94
5008	5964	0.84	2554	2989	0.85	1902	2151	0.88
4254	4529	0.94	2536	2771	0.92	1867	2037	0.92
3984	4392	0.91	2484	2791	0.89	1854	2018	0.92
3748	4139	0.91	2389	2733	0.87	1840	2108	0.87
3725	3938	0.95	2355	2657	0.89	1831	2040	0.90
3646	3970	0.92	2332	2723	0.86	1825	1994	0.92
3607	3901	0.92	2331	2574	0.91	1780	1989	0.89
3365	3758	0.90	2247	2405	0.93	1779	2163	0.82
3328	3614	0.92	2241	2506	0.89	1767	1868	0.95
3286	3773	0.87	2195	2329	0.94	1765	2071	0.85
3238	3470	0.93	2162	2563	0.84	1751	1814	0.97

**Table 2:** Granite rock block information.

Area ${cm}^{2}$	Convex hull cm^2^	Solidity	Area cm^2^	Convex hull cm^2^	Solidity	Area cm^2^	Convex hull cm^2^	Solidity
10,296	10,877	0.95	3778	4394	0.86	2434	2742	0.89
10,053	11,500	0.87	3643	4383	0.83	2420	2548	0.95
9542	10,393	0.92	3587	4183	0.86	2320	2652	0.87
8577	9579	0.90	3553	3934	0.90	2313	2563	0.90
6889	7875	0.87	3369	3784	0.89	2301	2483	0.93
6340	7412	0.86	3090	3411	0.91	2298	2684	0.86
6226	7117	0.87	3063	3510	0.87	2241	2418	0.93
6218	7230	0.86	3061	3494	0.88	2241	2684	0.83
4701	4990	0.94	3021	3318	0.91	2220	2383	0.93
4640	5188	0.89	3009	3350	0.90	2204	2527	0.87
4583	5027	0.91	2991	3142	0.95	2203	2480	0.89
4535	4970	0.91	2957	3348	0.88	2193	2428	0.90
4486	4746	0.95	2841	3177	0.89	2178	2469	0.88
4374	5098	0.86	2822	3021	0.93	2178	2585	0.84
4292	4856	0.88	2818	3008	0.94	2149	2425	0.89
4192	4645	0.90	2776	3086	0.90	2125	2456	0.87
4090	4514	0.91	2629	2892	0.91	2107	2417	0.87
3984	4246	0.94	2569	2823	0.91	2093	2258	0.93
3812	4201	0.91	2492	2952	0.84	2086	2426	0.86
3783	4051	0.93	2449	2600	0.94	2054	2369	0.87

From Fig. 11 can be seen in the graph of the larger rock solidity range of 0.82–0.97, mainly concentrated between 0.87 and 0.95; from Fig. 12 can be seen in the graph of the larger rock solidity range of 0.83–0.95, mainly concentrated between 0.85 and 0.95. The solidity of the contour of block is high, which can be concluded that the shape of the blasting rock blocks closes to convex polyhedron.

### The seed point marking method based on the solidity of rock block contour

**Figure 13:**
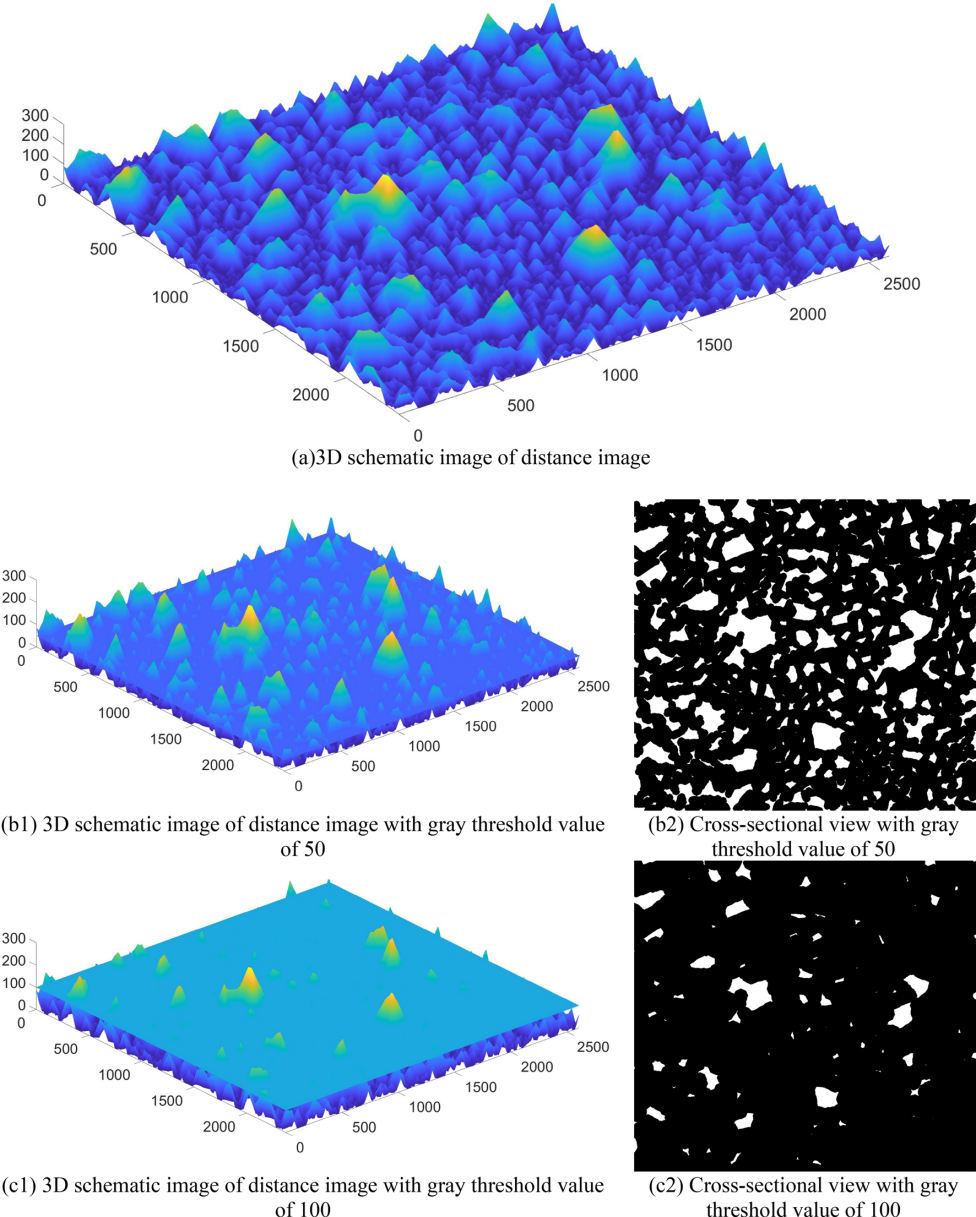
3D schematic image of distance image and 2D cross-sectional image under different gray thresholds.

Based on the above study, the seed point marking method based on the solidity of rock block contour is proposed on the basis of the distance transformation optimized watershed algorithm. The method makes the adhesion part (that is, adherent rock blocks in the blasted rock image) in the distance transformation image segmented by selecting an appropriate gray threshold, and calculates the solidity by image contour detection technology, and marks it as a seed point if the contour solidity is greater than the set solidity threshold. As shown in Fig. 13, (a) is a 3D schematic image of the distance image, and the height is the gray value of the corresponding coordinate; (b1) is a 3D schematic image of the distance image when the gray threshold value is 50, and (b2) is its corresponding 2D cross-section; (c1) is a 3D schematic image of the distance image when the gray threshold value is 100, and (c2) is its corresponding 2D cross-section.

When the grayness threshold is too small, although smaller blocks can be split and seed point marked, more large rock blocks are not divided, as can be seen in Fig. 13b1,b2. And most of the contour solidity is less than the solidity threshold value, which does not work as seed points. It can be seen from Fig. 13c1,c2 that when the gray threshold is too large, only the large size rock blocks can be segmented. Due to the large size difference, a single gray threshold does not satisfy the seed point marking of rock blocks, so multiple gray thresholds need to be processed and the solidity is calculated for the contours in the 2D image after each gray threshold is processed. If it is greater than the solidity threshold, the interior of the contour is filled and marked as a seed point.

**Figure 14:**
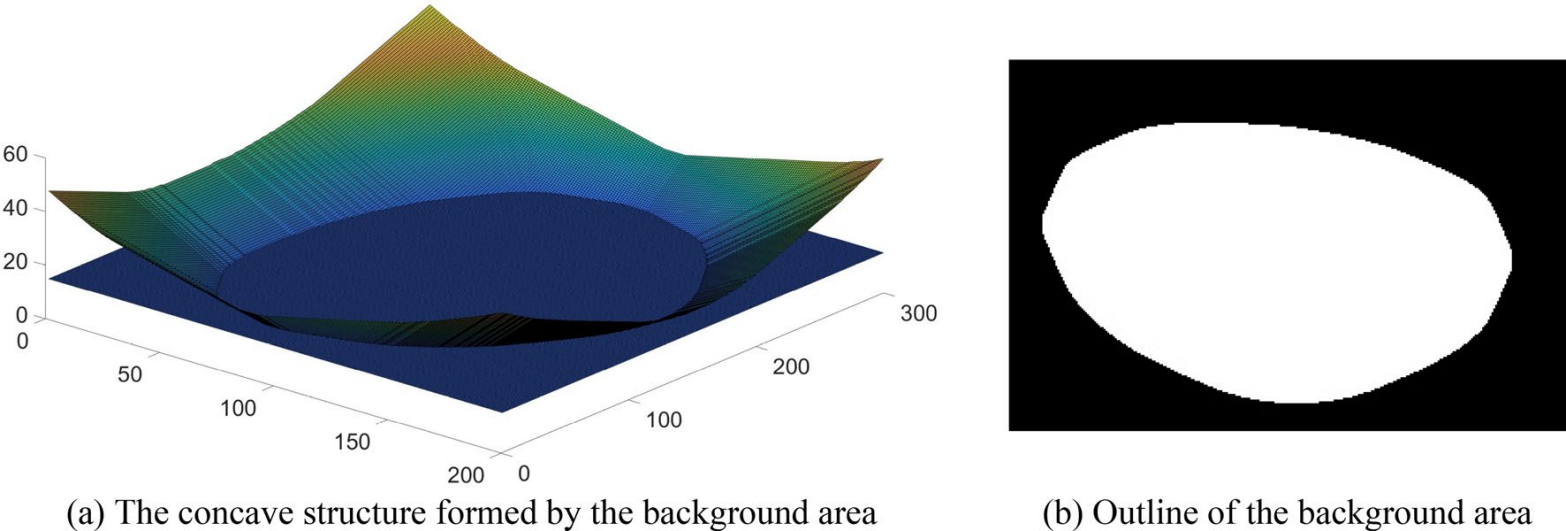
Recessed structure formed in the background area and 2D cross-sectional profile.

**Figure 15:**
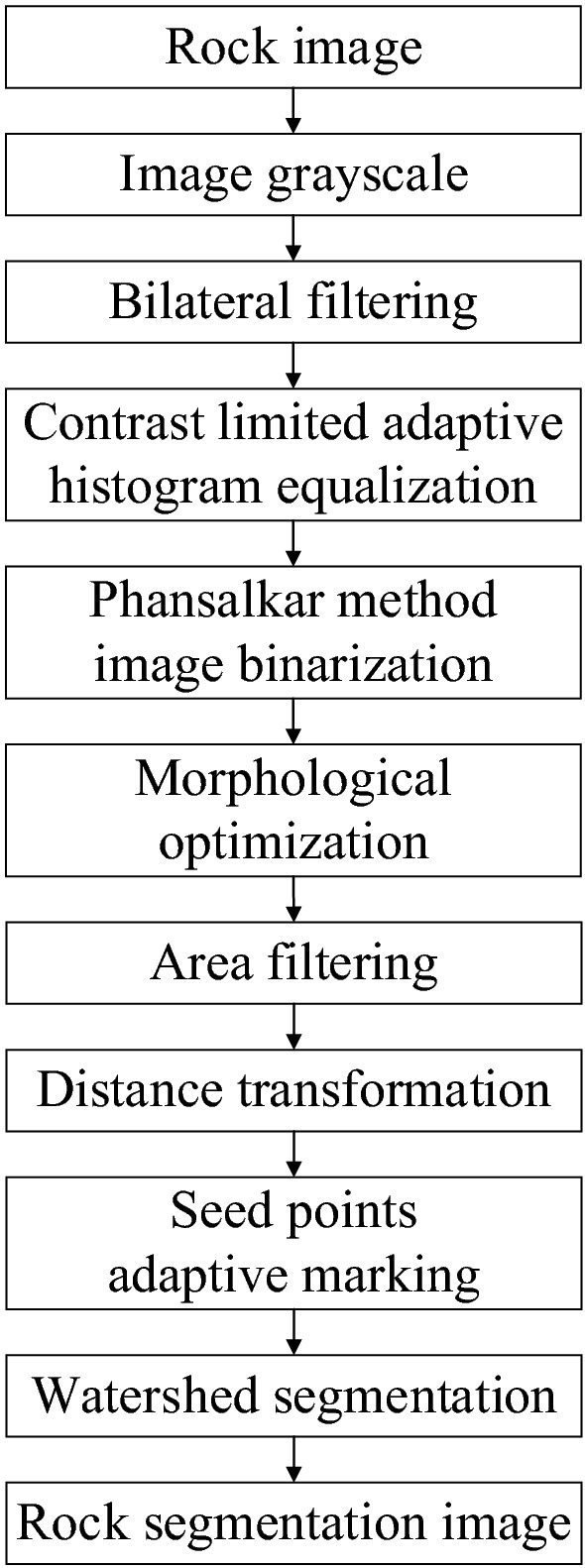
Flow chart of the proposed rock image segmentation method.

It should be noted that since this seed point marking method is based on the distance transform image, there may be two cases as follows: ① When the solidity of the contour of a background region in a binary image is too high, a depressed structure as in Fig. 14a will be formed in this background region after the distance transformation process, when the contour is taken using the gray threshold, a contour as in Fig. 14b will be formed, and the solidity of this contour is greater than the solidity threshold, which will cause wrong segmentation if the contour is marked as a seed point. ② As shown in the red marker in Fig. 15, this noise is caused by the wrong differentiation of Phansalkar. Obviously, morphological optimization and area filtering do not eliminate this noise, and the contour is smaller than the solidity threshold value. Therefore, when determining whether the contour is a seed point or not, it is necessary to determine whether there is a background area or noise within the contour, and if it exists, it is necessary to determine whether the contour is generated by the background area or noise.

The pseudo code of the seed point marking method based on the solidity of rock block contour is shown in steps 8–12 of algorithm 1.

### The adaptive watershed algorithm based on the solidity of rock block contours

Achieving accurate segmentation of blasted rock images requires a complete process, which mainly consists of image denoising, histogram equalization, image binarization, morphological optimization, distance transform, seed point marking and watershed segmentation. Based on the above research, an adaptive watershed segmentation algorithm is proposed for blasted rock piles images based on the solidity of rock contours. The method performs adaptive segmentation based on the gray feature of blasted rock piles image and the rock contour features without any manual intervention. The process is shown in Fig. 15, and algorithm 1 shows the main steps of the adaptive watershed algorithm based on the solidity of rock block contours.
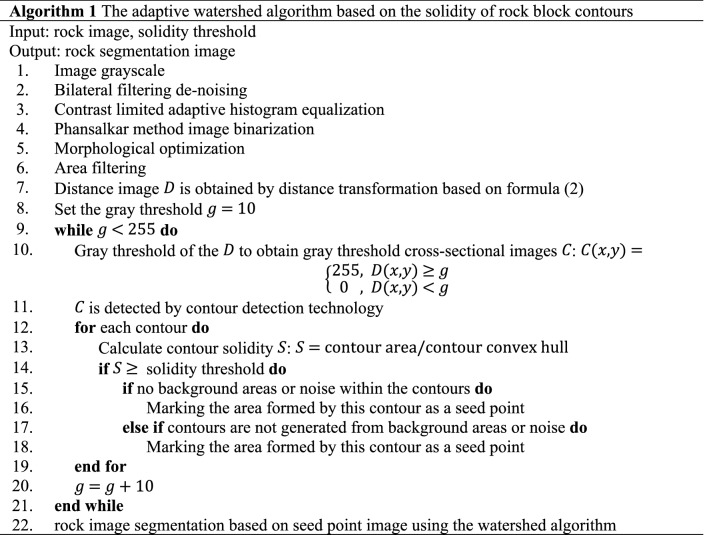


## Experimental results and analysis

### Seed point marking and segmentation results

**Figure 16:**
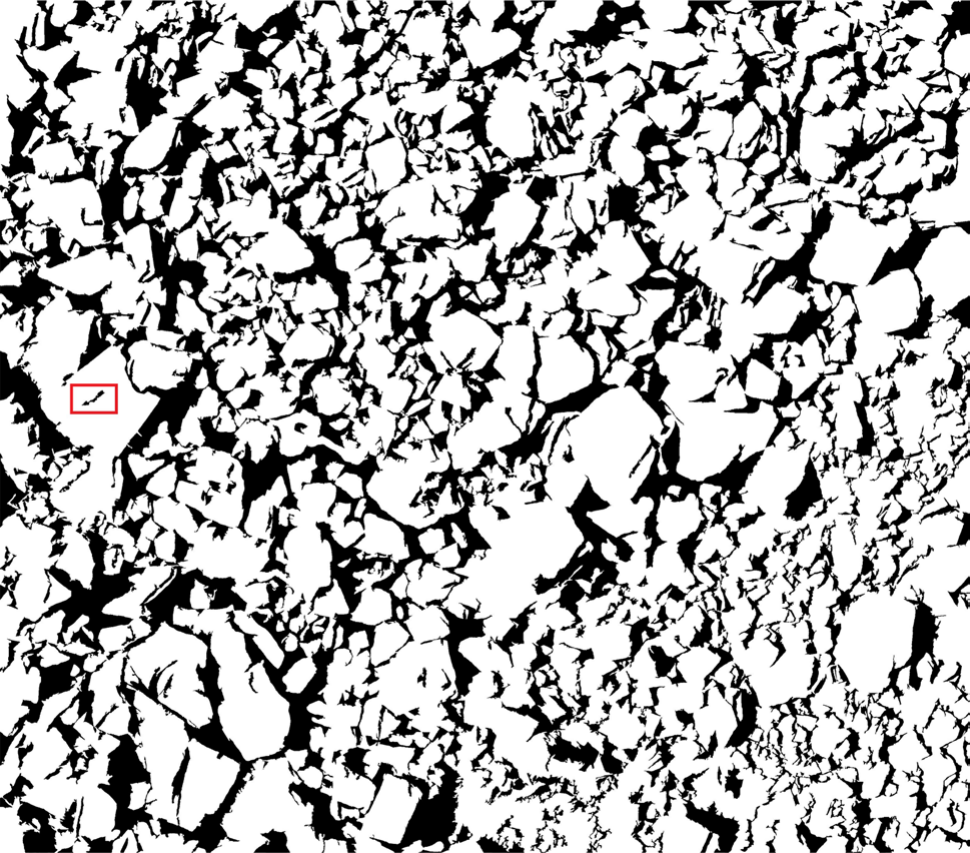
Granite blasted rock binary image.

**Figure 17:**
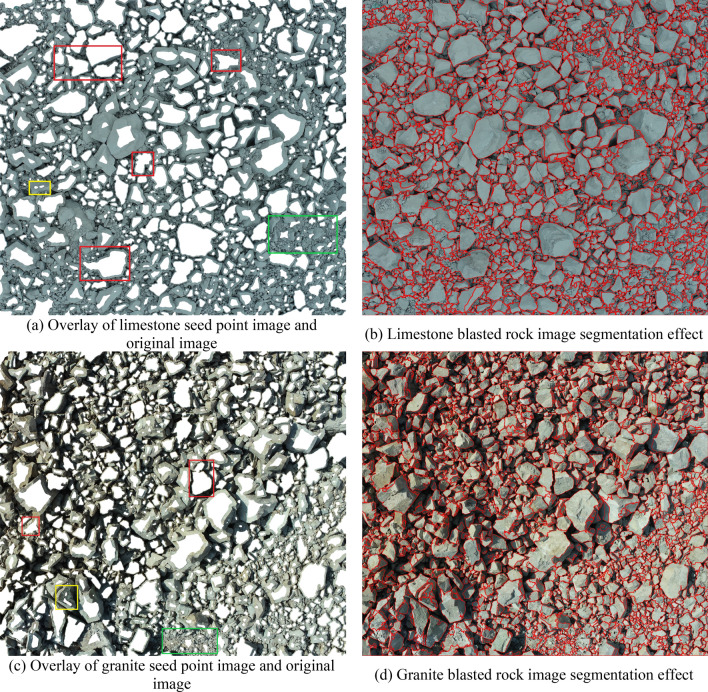
Blasted rock seed point marking effect and segmentation effect for a solidity threshold of 0.8.

**Figure 18:**
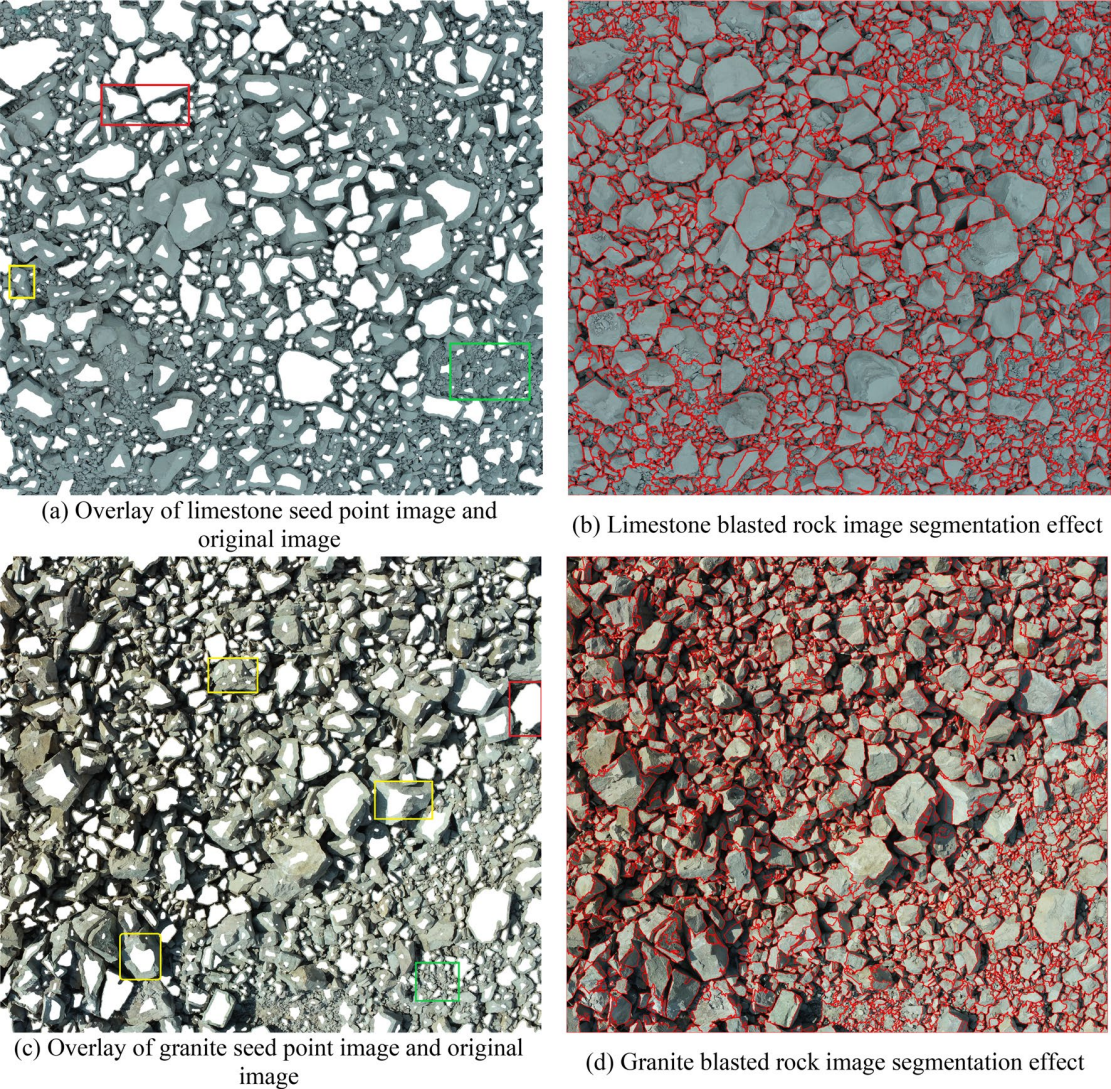
Blasted rock seed point marking effect and segmentation effect for a solidity threshold of 0.85.

**Figure 19:**
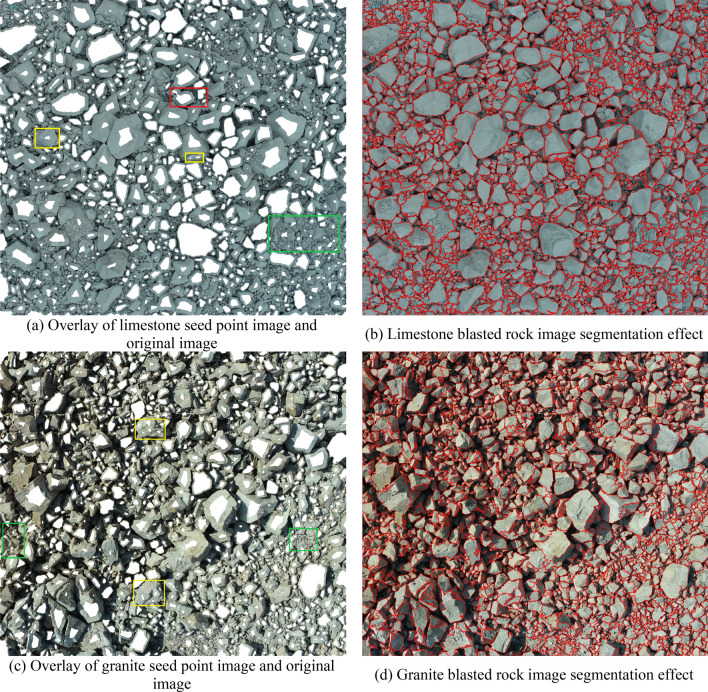
Blasted rock seed point marking effect and segmentation effect for a solidity threshold of 0.9.

Using the blasted rock block images of limestone and granite in Fig. 4 as the test objects, the applicability and accuracy of the adaptive watershed algorithm based on rock block contour solidity for blasted rock images are verified. Firstly, the images are preprocessed, and the binary images of limestone blasted rock is shown in Fig. 8a, and the binary images of granite blasted rock are shown in Fig. 16. Then the blasted rock images are segmented using the method proposed in this study. Figures 17, 18 and 19 show the effect of seed point labeling and segmentation with solidity threshold values of 0.8, 0.85, and 0.9, respectively.

### Analysis of seed point marking results

From the seed point images in Figs. 17, 18 and 19, it can be seen that the seed point marking method based on the solidity of rock block contours can effectively mark the rock blocks, especially the larger ones. Comparing the seed point image of the blasted rock piles with its pre-processed binary image, it is found that the seed point marking method can avoid the effect of noise inside the binary image on the segmentation, as shown in the red box in Fig. 16. However, the method also has some problems.

1. Some of the severely adhered blocks are not separated, as shown in the red boxes in the seed point images of Figs. 17, 18 and 19. It can be seen from the image that the problem is likely to arise when a large block heavily adheres to a small block and the difference in grayscale is small, due to the result that there are no local minima in the small block after the distance transformation and that solidity of the contours formed by the small and large rock blocks during seed point marking is greater than the solidity threshold.
2. Multiple seed point markings were performed on some of the rock blocks, as shown in the yellow boxes in the seed point images of Figs. 17, 18 and 19. From the figures, it can be seen that the rock blocks with multiple seed points fall into two main categories: the presence of multiple faces and large surface undulations in the image, which, due to natural light, cause the presence of shadows on the back of the block or lower areas of the surface, which affect the seed point marking.
3. There are no seed point markers for some of the smaller size rock blocks, as shown in the green boxes in the seed point plots of Figs. 17, 18 and 19. The main reason for this problem is the elimination of the background in the area of smaller rock blocks during morphological optimization and area filtering.

By comparing the seed point images with different solidity thresholds, it can be found that with the increase of solidity threshold, the case of no segmentation of the adherent rock block gradually decreases, but the case of multiple seed point marking of the rock block gradually increases, so it is necessary to select a suitable solidity threshold for seed point marking of the rock block to achieve the best segmentation effect.

### Analysis of image segmentation results of blasted rock piles

From the segmentation results in Figs. 17, 18 and 19, it can be seen that the adaptive watershed algorithm based on the solidity of rock block contours can achieve more accurate segmentation of the severely stacked and adhered blast rock images, especially the limestone blast rock images with less noise. In contrast, the segmentation effect of granite blast rock images is poor compared with that of limestone blast rock images due to the influence of rock block shadows and other problems. In order to evaluate the segmentation results with quantitative indexes, this study uses the manual segmentation image as the segmentation criterion to evaluate the segmentation effect of two blasted rock images, in which the manual segmentation image of limestone blasted rock is shown in Fig. 10a and the manual segmentation image of granite blasted rock is shown in Fig. 10b.

**Figure 20:**
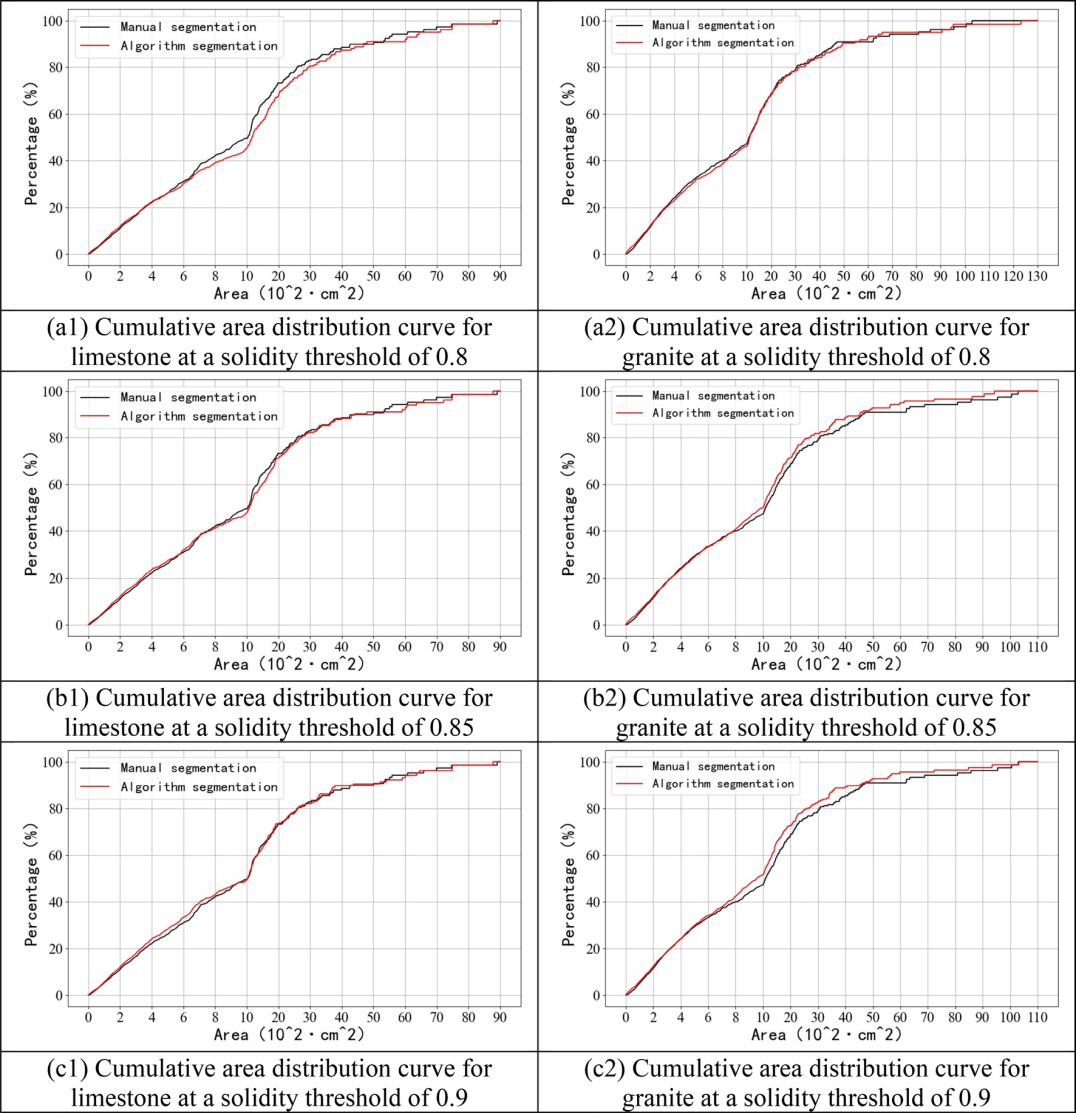
Cumulative area distribution curve.

Figure 20 show the cumulative distribution curves of the area of rock blocks in the images of limestone and granite blasted rock piles. The calculation formula is shown in Eq. (4).

4 $$P=\frac{{S}_{x}}{{S}_{total}},$$

where $P$ is the cumulative area ratio of rock blocks; ${S}_{total}$ is the sum of the areas of all identified rock blocks across the blasted rock image; and ${S}_{x}$ is the cumulative area of rock blocks in part $x$ of area classification.

As can be seen from Fig. 20, for rock segmentation of limestone blasted rock piles images, the agreement between the cumulative distribution curves of the area of the algorithm proposed in this study and the manual segmentation results gradually increases with the increase of the solidity threshold. but rock segmentation of granite blasted rock images becomes progressively worse as the solidity threshold increases. From Fig. 20c1, it can be seen that there are some differences in the segmentation results of limestone rocks with area ranges between 3600–4300 and 5500–6000 cm^2^. From Fig. 20a2, it can be seen that there are some differences in the segmentation results of granite rocks in the area range between 4000–5000 and 6000–7000 cm^2^, and the maximum area rock segmentation error is larger.

Table 3 gives a comparison of the three characteristic area parameters, ${Area}_{20}$, ${Area}_{50}$ and ${Area}_{80}$, at different solidity thresholds, which represent the areas corresponding to cumulative area distribution percentages of 20%, 50%, and 80% respectively. As can be seen from the table, like the solidity threshold increases, the ${Area}_{20}$ error for limestone gradually increases, while the ${Area}_{50}$ and ${Area}_{80}$ errors gradually decrease. The opposite is true for granite, where the ${Area}_{20}$ error decreases with increasing solidity threshold, while the ${Area}_{50}$ and ${Area}_{80}$ errors gradually increase.

**Table 3:** Three characteristic area parameters.

		Limestone			Granite		
		${Area}_{20}$	${Area}_{50}$	${Area}_{80}$	${Area}_{20}$	${Area}_{50}$	${Area}_{80}$
Manual segmentation		354.2	1034.1	2580.7	325.7	1084.7	3050.5
Proposed algorithm segmentation	Solidity threshold = 0.8	349.9	1163.5	2927.0	335.4	1112.4	3136.5
	Relative error ($\mathrm{\%}$)	− 1.21	12.5	13.4	2.97	2.53	2.82
	Solidity threshold = 0.85	340.2	1081.1	2689.5	329.3	1001.2	2651.8
	Relative error ($\mathrm{\%}$)	− 3.95	4.55	4.22	1.11	− 7.70	− 13.07
	Solidity threshold = 0.9	332.3	1020.8	2602.8	323.3	952.3	2556.9
	Relative error ($\mathrm{\%}$)	− 6.18	− 1.28	0.86	− 0.74	− 12.21	− 16.18
Literature^45^		494.3	1324.5	3240.0	386.9	1178.4	2871.9
Relative error (%)		39.55	28.08	25.55	18.79	8.64	− 5.85
Literature^46^		296.9	917.1	2489.9	294.4	958.3	2582.5
Relative error (%)		− 16.18	− 11.31	− 3.52	− 9.61	− 11.65	− 15.34

Tables 4 and 5 give the comparison of the number of rocks in different area zones for the image segmentation of limestone and granite blasted rock piles, respectively, and it should be noted that only the number of rocks with an area of 100 cm^2^ or more is counted. As can be seen from Table 4, the accuracy of block segmentation for limestone blasted rock images above 100 cm^2^ is above 95.80%. with the increase of solidity threshold, the number of rocks in the 100–1000 ${\mathrm{cm}}^{2}$ interval gradually increases, and the rocks above 1000 cm^2^ are less affected by the solidity threshold. Table 5 shows that the accuracy of rock segmentation for granite blasted block images above 100 cm^2^ is above 95.65%, and the number of rocks in the 100–1000 cm^2^ interval gradually increases with the increase of solidity threshold, while the number of rocks above 1000 cm^2^ gradually decreases. This is mainly due to the fact that as the solidity threshold increases, the area of large rock seed points gradually decreases and even splits into multiple seed points, resulting in an increase in the number of small rocks and a decrease or no change in the number of large rocks in the segmentation result, as shown in the seed point marking images in Figs. 18 and 19.

**Table 4:** The number of rock blocks in the image segmentation result of limestone blasted rock piles.

		100–max	100–1000	1000–3000	3000–max
Manual segmentation		904	750	132	22
Proposed algorithm segmentation	Solidity threshold = 0.8	872	715	132	25
	Relative error ($\mathrm{\%}$)	− 3.54	− 4.67	0.00	13.64
	Solidity threshold = 0.85	915	761	131	23
	Relative error ($\mathrm{\%}$)	0.22	1.47	− 0.76	4.55
	Solidity threshold = 0.9	942	787	131	24
	Relative error ($\mathrm{\%}$)	4.20	4.93	− 0.76	9.09
Literature^45^		780	604	148	28
Relative error (%)		− 13.2	− 19.47	12.12	27.27
Literature^46^		978	833	124	21
Relative error (%)		8.19	11.07	− 6.06	− 4.55

**Table 5:** The number of rock blocks in the image segmentation result of granite blasted rock piles.

		100–max	100–1000	1000–3000	3000–max
Manual segmentation		1196	1006	156	34
Proposed algorithm segmentation	Solidity threshold = 0.8	1144	946	164	34
	Relative error ($\mathrm{\%}$)	− 4.35	− 5.96	5.13	0.00
	Solidity threshold = 0.85	1184	993	160	31
	Relative error ($\mathrm{\%}$)	− 1.00	− 1.29	2.56	− 8.82
	Solidity threshold = 0.9	1221	1033	158	30
	Relative error ($\mathrm{\%}$)	2.09	2.68	1.28	− 11.76
Literature^45^		1161	950	178	33
Relative error (%)		− 2.93	− 5.57	14.10	− 2.94
Literature^46^		1301	1107	166	28
Relative error (%)		8.78	10.04	6.41	− 17.65

Therefore, among the three solidity thresholds, the best solidity threshold interval for limestone blasted rock image segmentation is 0.85–0.9, the best solidity threshold interval for granite blasted rock image segmentation is 0.8–0.85, considering the cumulative area distribution curve, characteristic area parameters and the number of rock blocks. In general, the algorithm proposed in this paper is consistent with the manual segmentation results under the condition that a suitable solidity threshold is selected.

### Comparison with current methods

**Figure 21:**
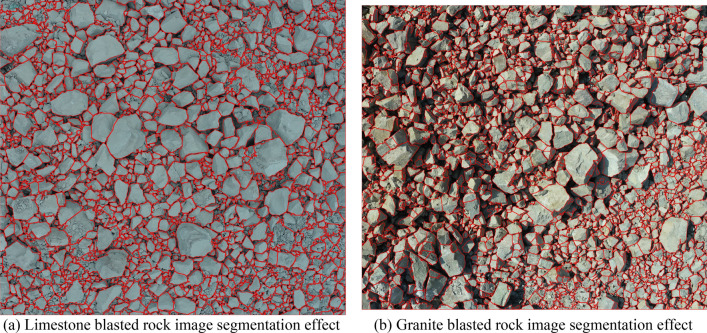
The segmentation effect using the segmentation method of the literature^45^.

**Figure 22:**
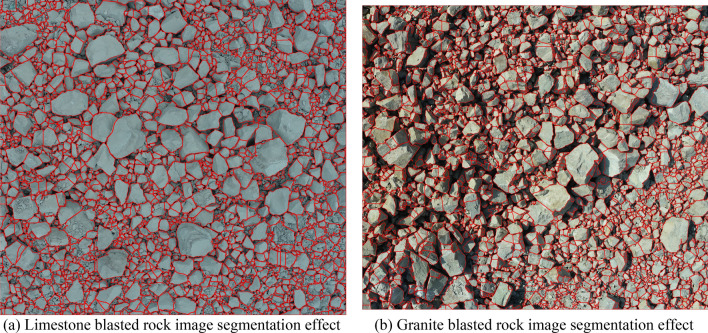
The segmentation effect using the segmentation method of the literature^46^.

**Figure 23:**
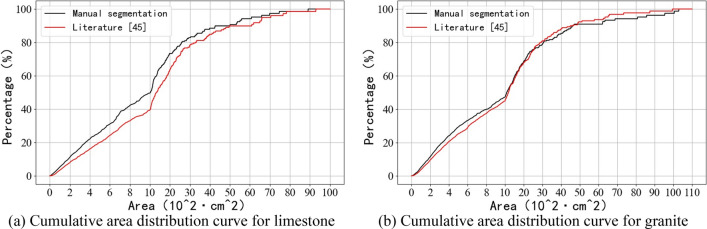
The cumulative area distribution curve for segmentation results in the literature^45^.

**Figure 24:**
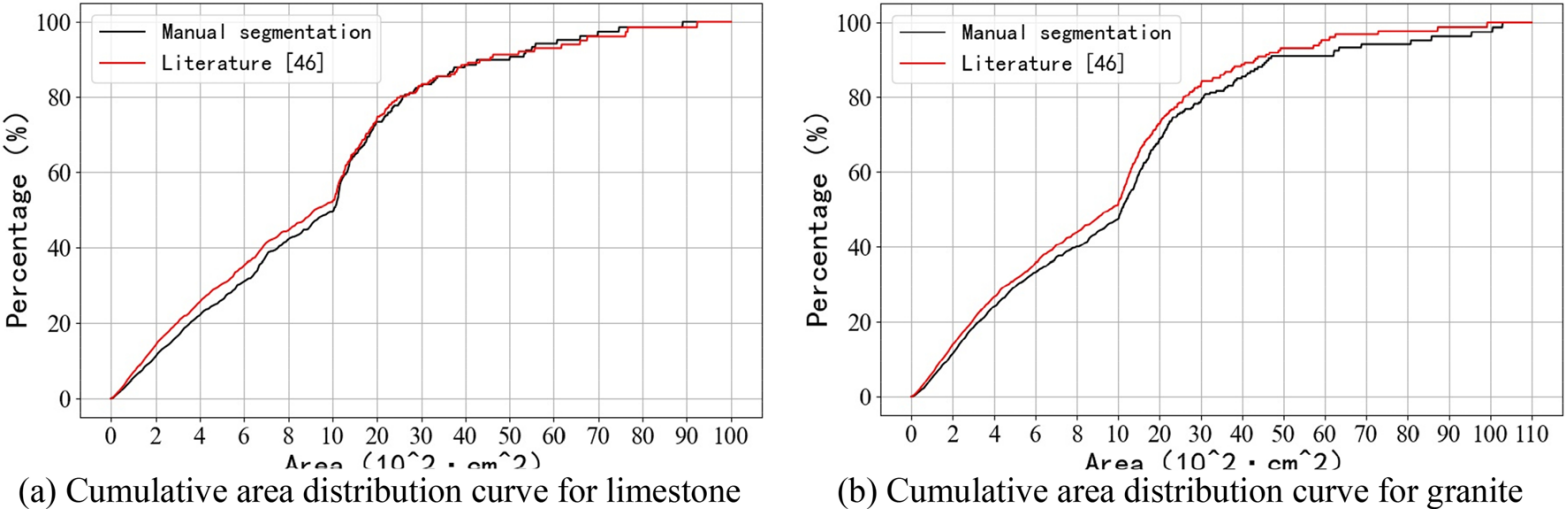
The cumulative area distribution curve for segmentation results in the literature^46^.

To evaluate the performance of the algorithm proposed in this study, the algorithm was compared with two other watershed improvement methods for rock segmentation, as described in the literature^45,46^, respectively. Figures 21 and 22 show the segmentation results using the segmentation methods from the literature^45,46^, respectively. Figures 23 and 24 show the cumulative area distribution curve of the literature^45,46^ segmentation results, respectively. The three characteristic area parameters, ${Area}_{20}$, ${Area}_{50}$ and ${Area}_{80}$, are shown in Table 3. The comparison of the number of rocks in different area for the segmentation of limestone and granite blasted rock images is shown in Tables 4 and 5, respectively.

As can be seen from Figs. 23a and 24a, the method of literature^46^ gives better results for limestone compared to the method of literature^45^, with cumulative area distribution curve over 1000 cm^2^ is more similar to the result of manual segmentation. As can be seen from Figs. 23b and 24b, the results of the method of literature^45^ are better than those of literature^46^ for granite. From Tables 3, 4 and 5, it can be seen that among the three segmentation methods, the method proposed in this study has the best segmentation results, and its segmentation results will be closer to the manual segmentation after selecting a suitable solidity threshold.

## Conclusion

In this study, to avoid errors caused by rock block overlap, image acquisition of blasted rock piles is performed using tilt photogrammetry. Then, the accurate rock target area was obtained through the image pre-processing process. Finally, the binary image is segmented using the adaptive watershed segmentation algorithm of blasted rock image based on rock block shape. This method enables automatic segmentation of blasted rock piles rock blocks is achieved. We compared the performance of the proposed method with the manual sieving results and with three methods in the literature^45,46^. The main conclusions are as follows:

1. The pre-processing process of blasted rock image and its influence on the segmentation results are described, and the Phansalkar method is introduced. By comparing the results with the Otsu method, it is proved that the method is more applicable to the binarization of blasted rock images.
2. The principle of the watershed algorithm based on distance transformation is described, and the reasons why the algorithm is prone to serious over-segmentation are analyzed. The study revealed a high-solidity of the blasted rock block contour by analyzing the shape of blocks, a seed point marking method based on the solidity of rock block contours is proposed, and forms an adaptive watershed segmentation algorithm for blasted rock images based on block shapes. The algorithm solves the problem of mis-segmentation of blasted rock images caused by severe adhesion and large differences in particle size.
3. The algorithm can effectively mark the seed points of blasted rock blocks and avoid the effect caused by noise inside the rock blocks of binary images, the segmentation results are highly similar to the area cumulative distribution curve of the manual segmentation results, and the errors of the different indexes are smaller than the segmentation methods adopt in the literature^45,46^, which proves the effectiveness and superiority of the algorithm for rock block segmentation of blasted rock piles images.

The adaptive watershed algorithm based on the solidity of rock block contours proposed in this study considers the contour properties of rock blocks in the segmentation, resulting in a more significant improvement in segmentation accuracy. However, it should be acknowledged that the method requires different solidity thresholds for different block image segmentation to achieve the best segmentation results. In future research, solidity thresholds for more kinds of rock will be investigated and determined, making the proposed method more widely applied.

## Data Availability

All data used to support the findings of this study are available from the corresponding author upon request.
